# The relationships between the plasma metabolome and orthostatic blood pressure responses

**DOI:** 10.1038/s41598-023-44226-z

**Published:** 2023-10-25

**Authors:** Christian Zambach, Jingxue Pan, Sofia Gerward, Artur Fedorowski, J. Gustav Smith, Gunnar Engström, Viktor Hamrefors

**Affiliations:** 1 Department of Clinical Sciences, Clinical Research Center, Lund University, Box 50332, 20313 Malmö, Sweden; 2 Department of Internal Medicine, Skåne University Hospital, Lund, Sweden; 3 Division of Child Healthcare, Department of Pediatrics, Tongji Hospital, Tongji Medical College, Huazhong University of Science and Technology, Wuhan, China; 4 Department of Cardiology, Karolinska University Hospital, Stockholm, Sweden; 5 Department of Medicine, Karolinska Institutet, Stockholm, Sweden; 6 Department of Cardiology, Clinical Sciences, Lund University and Skåne University Hospital, Lund, Sweden; 7 Wallenberg Center for Molecular Medicine and Lund University Diabetes Center, Lund University, Lund, Sweden; 8 The Wallenberg Laboratory/Department of Molecular and Clinical Medicine, Institute of Medicine, Gothenburg University, Gothenburg, Sweden; 9 Department of Cardiology, Sahlgrenska University Hospital, Gothenburg, Sweden; 10 Department of Cardiology, Skåne University Hospital, Malmö, Sweden

**Keywords:** Cardiovascular diseases, Biomarkers

## Abstract

Whereas autonomic dysfunction and the metabolic syndrome are clinically associated, the relationships with the plasma metabolome is unknown. We explored the association between orthostatic blood pressure responses and 818 plasma metabolites in middle-aged subjects from the general population. We included 3803 out of 6251 subjects (mean age, 57 years; 52% women) from the Malmö sub-cohort of The Swedish CardioPulmonary bioImage Study with information on smoking habits, diabetes, antihypertensive drug treatment, anthropometrics, hemodynamic measurements and 818 plasma metabolites (mass-spectrometry). The associations between each metabolite and orthostatic systolic blood pressure responses were determined using multivariable linear regression analysis and *p* values were corrected using the Bonferroni method. Six amino acids, five vitamins, co-factors and carbohydrates, nine lipids and two xenobiotics were associated with orthostatic blood pressure after adjusting for age, gender and systolic blood pressure. After additional adjustments for BMI, diabetes, smoking and antihypertensive treatment, the association remained significant for six lipids, four amino acids and one xenobiotic. Twenty-two out of 818 plasma metabolites were associated with orthostatic blood pressure responses. Eleven metabolites, including lipids in the dihydrosphingomyelin and sphingosine pathways, were independently associated with orthostatic systolic blood pressure responses after additional adjustment for markers of cardio-metabolic disease.

## Introduction

Orthostatic hypotension (OH), defined as decrease of systolic and/or diastolic blood pressure of more than 20/10 mmHg upon standing^1,2^, is a common condition in the population. Whereas there are some specific reversible clinical causes of this condition, such as excessive blood pressure lowering therapy or dehydration, OH in the population often indicates some degree of cardiovascular autonomic dysfunction^3^. OH is strongly associated with cardiovascular disease (CVD) and often correlates with markers of poor cardio-metabolic health. Previous studies have shown that OH, or even more subtle changes indicating cardiovascular autonomic dysfunction, can be an early sign of cardio-metabolic disease and predict cardiovascular mortality^4,5^.

The metabolic syndrome, defined by visceral obesity, dyslipidemia, hyperglycemia and hypertension^6^, is a major health concern that has been affecting a steadily increasing part of the global population. Those affected are known to be at high risk of cardiovascular events such as stroke and myocardial infarction that account today for approximately one third of deaths globally^5,7,8^. Unhealthy diet and a low level of physical activity are two major risk factors that can be modified.

In recent decades the development of methods to identify and measure quantities of metabolites has accelerated^9^. The metabolome can be seen as a footprint of our overall health status and its composition may potentially yield valuable information on multiple diseases. It is affected by genetics, diet, physical activity, the microbiome, inflammation/stress, the metabolic condition, hormonal homeostasis and drug treatment^10^. A special attention has been paid to the composition of metabolome in order to investigate if certain single metabolites, or compositions of metabolome can be linked to cardio-metabolic disease in a way that could make them suitable as markers in risk assessment^11–15^.

In recent years, bacterial endotoxins like lipopolysaccharides (LPS), degradants of components of red meat such as trimethylamine oxide (TMAO), several short chain fatty acids (SCFA), branched-chain amino acids (BCAA), aromatic amino acids (AAA) as well as bile acids were found to be associated with different outcomes of cardio-metabolic disease^11–15^.

Besides changes in lifestyle and diet, fecal microbiota transplantation, probiotic, prebiotic and antibiotic treatment as well as small molecule antimicrobial enzyme therapeutics have been proposed as interventions that target the gut microbiota and thereby imbalances in the metabolome.

Whether components of the metabolome are associated with markers of autonomic dysfunction, such as impaired orthostatic blood pressure responses, is unknown.

Orthostatic hypotension is closely associated with cardiovascular risk factors and the metabolic syndrome, especially in the elderly population. We therefore hypothesized that metabolites, such as for example sphingolipids, that are associated with hypertension, diabetes and cardiovascular disease, may also be associated with cardiovascular autonomic dysfunction in terms of a maladaptive orthostatic blood pressure response.

In this explorative study we examined the association between orthostatic blood pressure responses and untargeted plasma metabolites in a random sample from the middle-aged population.

## Methods

### Study population

The Swedish CardioPulmonary bioImage Study (SCAPIS) is a collaborative study between six Swedish universities and university hospitals (Gothenburg, Malmö, Linköping, Stockholm, Uppsala and Umeå). A total of 30,154 subjects from the general population in the age range 50–64 years were invited by letter to participate during the period 2013–2018^16^.

**Figure 1:**
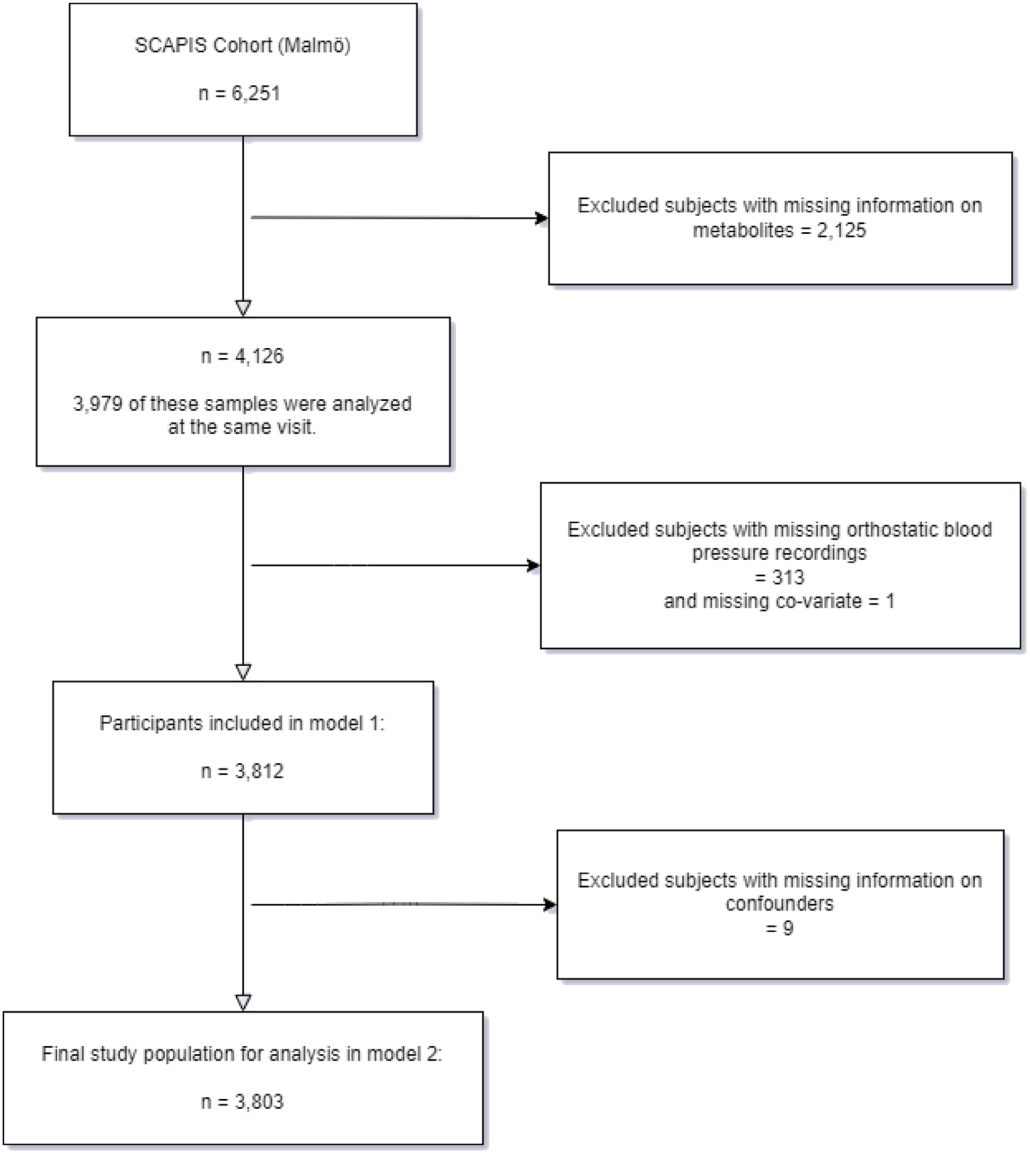
Flowchart of the study population.

A total of 6251 men and women were included at the Malmö site, which corresponded to a participation rate of 53%. Comprehensive examinations were carried out at three different days with one week apart. Of the 6251 participants, a total of 4126 individuals had information on plasma metabolites measures, 3979 of these had recorded orthostatic blood pressure responses at the same visit. After excluding subjects with missing values of orthostatic blood pressure reaction (n = 313) and information on covariates (e.g., BMI, diabetes, smoking status, anti-hypertensive medicine, resting heart rate), a total of 3803 subjects were included in the fully adjusted analyses (Fig. 1).

Written informed consent was obtained from all participants in this study. The SCAPIS project was approved by the ethical review board of Umeå University and by the ethical review board in Lund (LU 2016/1031). All methods were performed in accordance with the relevant guidelines and regulations.

### Analysis of plasma metabolites

During the baseline examinations of SCAPIS, overnight fasting blood samples were drawn in the morning and then were stored at -80 ˚C until time of metabolomics analyses. In the study, non-targeted metabolomics (Metabolon Inc., Durham, North Carolina, USA) were performed on plasma samples. Samples were prepared by the automated MicroLab STAR® system from Hamilton Company (Reno, Nevada, USA). For the purposes of quality control, several internal standards were used prior to the extraction process. Proteins were precipitated with methanol and vigorously shaken for 2 min followed by centrifugation (Glen Mills GenoGrinder 2000) to remove proteins, dissociate small molecules bound to or trapped in the precipitated protein matrix, and to recover chemically diverse metabolites. The resulting extract was divided into five fractions: one for analysis by reverse phases (RP)/Ultrahigh Performance Liquid Chromatography-Tandem Mass Spectroscopy (UPLC-MS/MS) methods with negative ion mode electrospray ionization (ESI), one for analysis by HILIC/UPLC-MS/MS with negative ion mode ESI, two for analysis by two separate RP/UPLC-MS/MS methods with positive ion mode ESI, and one sample was reserved for backup. Raw data of metabolites were extracted, peak-identified and quality control-processed by Metabolon’s hardware and software which were described previously^17^. The compounds were annotated by matching to a library of more than 3300 purified standard metabolites or recurrent unknown entities. The library is based on authenticated standards that contains the retention time/index (RI), mass to charge ratio (m/z), and chromatographic data^17^. The missing values for each metabolite was imputed from the minimum value across all batches. In addition, each metabolite was assigned to broad biochemical classes, as well as to its specific biochemical class (metabolite sub-pathway). In our analyses, metabolites which were classified as drugs in the xenobiotics, were converted to present or absent. The metabolite levels were normalized by natural logarithm and the values were given in arbitrary units. Only annotated metabolites with a call rate > 75% and non-drug related xenobiotics were included in the analyses, resulting in 818 metabolites included, 96 of which were non-drug related xenobiotics.

### Orthostatic blood pressure reaction measurements

Orthostatic blood pressure reactions were calculated by subtracting the blood pressure after 3 min of standing up from the blood pressure measured after 5 min resting in the supine position measured in millimeter quicksilver (mmHg)^16^.

Orthostatic hypotension (OH) was defined as decrease in systolic and/or diastolic blood pressure greater than 20/10 mmHg upon standing^1,2^.

The blood pressure was measured twice in supine position in both arms with an automatic device after 5 minutes of rest (Omron M10‐IT. Omron Healthcare Co. Kyoto. Japan). The average of the two blood pressure recordings in the arm with the higher mean blood pressure was included in the analysis. For the recordings in the supine position, the arm was supported at heart level.

### Risk factor examinations

Baseline information was collected during examinations. Body weight (kg) and standing height (meter) were measured without shoes. Body mass index (BMI) was calculated as weight in kilograms divided by the square of height in meters (kg/m^2^). Smoking status was obtained from questionnaires and ascertained as smokers and non-smokers. Diabetes was defined as previously known diabetes (self-reported) or by getting diagnosed during the examinations by an elevated fasting plasma glucose sample. Antihypertensive treatment was defined according to the answer of the questionnaire: “Have you been taking medicines for high blood pressure in the last 2 weeks?”. Detailed information on the methods used in the Malmö cohort of SCAPIS are found elsewhere^16^.

### Statistical analyses

Continuous variables are presented as means ± standard deviations (SDs) and categorical variables are shown as numbers, percentages (n, %). The associations between each metabolite (independent variable, tested one by one) and orthostatic systolic blood pressure responses (dependent variable) were determined using multiple linear regression analysis. The multivariate models were adjusted for the potential confounders as follows: Model 1 was adjusted for age, gender and systolic blood pressure; Model 2 was additionally adjusted for BMI, diabetes, smoking status, and anti-hypertensive drug treatment.

Beta coefficients with 95% confidence intervals (Cis) are reported. Considering the number of metabolites (n = 818), we decided to use the Bonferroni correction to minimize the chance of false-positive findings^18^. A Bonferroni corrected *p* value of maximum 6.11 × 10^–5^ (0.05/818) was considered as significant in the analyses of metabolites in model 1. Then we selected the metabolites significantly associated with orthostatic systolic blood pressure responses in model 1, to examine their relationships with additional adjustments in model 2. A Bonferroni-corrected *p* value < 2.273 × 10^–3^ (0.05/22) was considered as significant in model 2. In addition, we also explored the associations between all metabolites and manifest OH using logistic regression analysis. The same two models were conducted and the results are reported as OR with 95% CI. A *p* value of < 6.11 × 10^–5^ was regarded as statistically significant.

All the analyses were performed using IBM SPSS Statistics V.27 (www.spss.com) and R studio (Version 1.3.1093).

## Results

### Characteristics of the study population

Characteristics of the study population are presented in Table 1. In short, 47.9% of the total 3812 participants were men. Mean age was 57 ± 4 years. On average, the study population had slightly elevated BMI (BMI 27 ± 4 kg/m^2^). Diabetes Mellitus was prevalent in 8.3%. Manifest orthostatic hypotension (OH) was present in 56 of 3803 recordings i.e. 1.5%. Systolic blood pressure was normal on average (122 ± 16 mmHg). A total of 14.4% were current smokers and 20.3% reported antihypertensive drug treatment.

**Table 1:** Characteristics of the study population.

Total number of subjects	3812
Prevalence of orthostatic hypotension (n, %)	56, 1.5
Age (years)	57.42 ± 4.27
Women (n, %)	1987, 52.1
BMI (Kg/m^2^)*	27.18 ± 4.43
Orthostatic systolic blood pressure decrease (mmHg)	− 4.40 ± 10.20
Supine systolic blood pressure (mmHg)	121.73 ± 16.14
Resting heart rate (bpm)	60.84 ± 9.04
Diabetes (n, %)*	314, 8.3
Smokers (n, %)*	548, 14.4
Anti-hypertensive drug treatment (n, %)*	772, 20.3

The values were presented as mean ± standard deviation (SD) for continuous variables and numbers, percentages (n, %) for categorical variables.

*This analysis was performed on 3803 participants.

Subjects that were excluded from the fully adjusted analyses (n = 2448) had a slightly higher prevalence of OH, higher resting blood pressure, and were slightly more likely to have diabetes and to be smokers (Supplementary Table 1).

### Orthostatic systolic blood pressure responses and plasma metabolites

**Figure 2:**
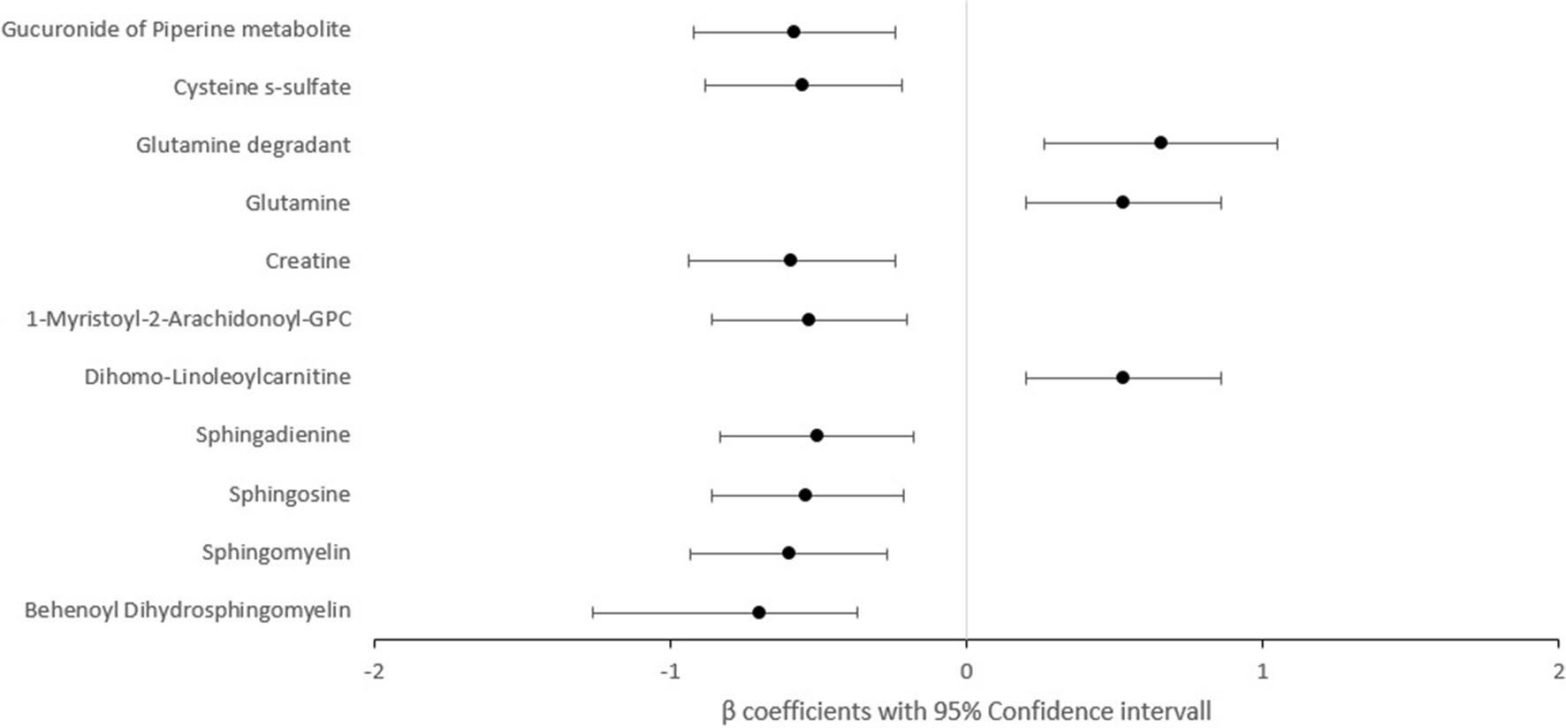
Forest plot on the significant associations between metabolites and systolic orthostatic blood pressure responses with full adjustment. The model was adjusted for age, gender, systolic blood pressure, diabetes, anti-hypertensive medication, BMI and smoking.

Significant results on associations between metabolites and orthostatic systolic blood pressure responses are depicted in Tables 2, 3 and Fig. 2. Adjusted for age, gender, systolic blood pressure (Model 1), we found six amino acids, five cofactors, vitamins and carbohydrates, nine lipids and two xenobiotics that were significantly associated with orthostatic blood pressure responses (Bonferroni corrected *p* value ≤ 6.11 × 10^–5^).

**Table 2:** Metabolites associated with orthostatic blood pressure responses.

	Chemical name	Super pathway	Sub-pathway	β coff.	95% CI		*P* value
					Lower limit	Upper limit	
1	Behenoyl Dihydrosphingomyelin	Lipid	Dihydrosphingomyelins	− 0.94	− 1.26	− 0.61	1.92 × 10^–8^
2	Aspartate	Amino acid	Alanine and Aspartate metabolism	− 0.87	− 1.21	− 0.54	3.74 × 10^–7^
3	Creatine	Amino acid	Creatine metabolism	− 0.86	− 1.21	− 0.51	1.22 × 10^–6^
4	Sphingomyelin	Lipid	Dihydrosphingomyelins	− 0.79	− 1.12	− 0.47	1.93 × 10^–6^
5	Sphingosine	Lipid	Sphingosines	− 0.78	− 1.10	− 0.45	2.51 × 10^–6^
6	Metabolonic Lactone sulfate	Partially characterized molecule	Partially characterized molecule	− 0.80	− 1.13	− 0.46	2.84 × 10^–6^
7	Cysteine s-sulfate	Amino Acid	Methionine, Cysteine, SAM and Taurine metabolism	− 0.79	− 1.12	− 0.46	3.26 × 10^–6^
8	Carotene Diol	Cofactors and vitamins	Vitamin A metabolism	0.78	0.45	1.11	3.77 × 10^–6^
9	Sphingadienine	Lipid	Sphingolipid synthesis	− 0.73	− 1.05	− 0.41	8.69 × 10^–6^
10	Asparagine	Amino acid	Alanine and Aspartate metabolism	0.73	0.40	1.05	1.22 × 10^–5^
11	Glutamine	Amino acid	Glutamate metabolism	0.72	0.40	1.05	1.34 × 10^–5^
12	Bilirubin (E,Z or Z,E)	Cofactors and vitamins	Hemoglobin and Porphyrin metabolism	0.72	0.39	1.05	1.65 × 10^–5^
13	Bilirubin (Z,Z)	Cofactors and vitamins	Hemoglobin and Porphyrin metabolism	0.72	0.39	1.05	1.69 × 10^–5^
14	Sphingomyelin)	Lipid	Dihydrosphingomyelins	− 0.71	− 1.04	− 0.38	2.2 × 10^–5^
15	1-stearoyl-2-oleoyl-GPE	Lipid	Phosphatidylethanolamine (PE)	− 0.71	− 1.03	− 0.38	2.22 × 10^–5^
16	Glucuronide of Piperine metabolite	Xenobiotics	Food component/Plant	− 0.71	− 1.04	− 0.38	2.53 × 10^–5^
17	Glycoursodeoxycholic acid sulfate	Lipid	Secondary Bile Acid metabolism	− 0.71	− 1.05	− 0.37	3.78 × 10^–5^
18	Dihomo-Linoleoylcarnitine	Lipid	Fatty acid metabolism (Acyl Carnitine, Polyunsaturated)	0.69	0.36	1.02	4.2 × 10^–5^
19	1-Myristoyl-2-Arachidonoyl-GPC	Lipid	Phosphatidylcholine (PC)	− 0.69	− 1.02	− 0.36	4.28 × 10^–5^
20	Glutamine degradant	Partially characterized molecule	Partially characterized molecule	0.82	0.42	1.21	4.51 × 10^–5^
21	Phenylacetate	Amino acid	Phenylalanine metabolism	0.68	0.35	1.00	4.96 × 10^–5^
22	2-Naphthol Sulfate	Xenobiotics	Chemical	− 0.67	− 0.99	− 0.34	5.39 × 10^–5^

Model 1: Adjusted for age, gender and supine systolic blood pressure.

**Table 3:** Metabolites associated with orthostatic blood pressure responses in fully adjusted models.

	Chemical name	Super pathway	Sub-pathway	β coff.	95% CI		*P* value
					Lower limit	Upper limit	
1	Behenoyl Dihydrosphingomyelin	Lipid	Dihydrosphingomyelins	− 0.70	− 1.26	− 0.37	3.42 × 10^–5^
2	Sphingomyelin	Lipid	Dihydrosphingomyelins	− 0.60	− 0.93	− 0.27	3.58 × 10^–4^
3	Sphingosine	Lipid	Sphingosines	− 0.54	− 0.86	− 0.21	1.28 × 10^–3^
4	Sphingadienine	Lipid	Sphingosines	− 0.50	− 0.83	− 0.18	2.27 × 10^–3^
5	Dihomo-Linoleoylcarnitine	Lipid	Fatty acid metabolism (Acyl Carnitine, polyunsaturated)	0.53	0.20	0.86	1.84 × 10^–3^
6	1-Myristoyl-2-Arachidonoyl-GPC	Lipid	Phosphatidylcholine (PC)	− 0.53	− 0.86	− 0.20	1.57 × 10^–3^
7	Creatine	Amino acid	Creatine metabolism	− 0.59	− 0.94	− 0.24	1.04 × 10^–3^
8	Glutamine	Amino acid	Glutamate metabolism	0.53	0.20	0.86	1.72 × 10^–3^
9	Glutamine degradant	Partially characterized molecule	Partially characterized molecules	0.66	0.26	1.05	1.08 × 10^–3^
10	Cysteine s-sulfate	Amino acid	Methionine, Cysteine, SAM and Taurine metabolism	− 0.55	− 0.88	− 0.22	1.24 × 10^–3^
11	Gucuronide of Piperine metabolite	Xenobiotic	Food component/plant	− 0.58	− 0.92	− 0.24	7.24 × 10^–4^

Model 2: Adjusted for age, gender, supine systolic blood pressure, diabetes mellitus, anti-hypertensive medication, BMI and smoking.

After additional adjustments for BMI, diabetes, smoking and antihypertensive treatment (Model 2, in Table 3), we found significant associations for six lipids, three amino acids, one xenobiotic and one uncharacterized molecule and orthostatic systolic blood pressure responses (Bonferroni corrected *p* value < 2.273 × 10^–3^).

Most metabolites had negative beta coefficients in relation to systolic blood pressure decrease (see Table 2, 3 and Fig. 2), i.e. higher levels of metabolites were associated with a less pronounced systolic blood pressure decrease. No significant associations were found between the levels of the metabolites and manifest orthostatic hypotension (data not shown). The respective molecules and the biological pathways in which the metabolites are included are listed in detail in Tables 2 and 3.

## Discussion

### Overall findings

We here performed an exploratory analysis of the possible associations between 818 metabolites and orthostatic blood pressure reactions in the general population. We found that eleven of the 818 metabolites were associated with systolic orthostatic blood pressure responses after adjusting for multiple testing and conventional cardiovascular risk factors. No metabolite was significantly associated with manifest OH after Bonferroni adjustment in this population.

Looking specifically at the relationships, 22 metabolites, half of them lipids, were associated with systolic orthostatic blood pressure responses when adjusted for age, gender and systolic blood pressure. Of these, eleven metabolites, likewise predominantly metabolites in lipid pathways, remained significantly associated when adjusting also for diabetes, BMI, smoking and anti-hypertensive treatment.

Lipids and xenobiotics that were associated with orthostatic blood pressure responses mainly showed a negative beta coefficient in relation to orthostatic blood pressure responses (i.e. higher levels were associated with less pronounced systolic blood pressure decrease) whereas vitamins, cofactors and most amino acids showed positive beta coefficients in relation to orthostatic blood pressure responses (i.e. higher levels of these were associated with more pronounced systolic blood pressure decrease on standing; Tables 2, 3 and Fig. 2).

The eleven metabolites that displayed significant associations with orthostatic blood pressure responses after adjusting for CVD risk factors were diverse. However, these included six lipids involved mainly in the dihydro-sphingomyelins, sphingosines, phosphatidylcholine (PC), and fatty acid metabolism (acyl carnitine, polyunsaturated) pathways. Furthermore, of 3 amino acids that play a role in the creatine, the glutamate and the methionine, cysteine, SAM and taurine metabolism pathways, and a food/plant based component (gucuronide of piperine metabolite C_17_H_21_NO_3_) as well as a glutamine degradant, respectively (Table 3).

### Lipid metabolites in relation to orthostatic blood pressure reactions

The six lipid metabolites that were associated with systolic orthostatic blood pressure responses are mainly sphingolipids, or their metabolites, that are found in cell membranes.

Behenoyl dihydro-sphingomyelin (DHSM) is a sphingolipid that is found in 5–10% of cells and plays a role in membrane-related biological processes^19^. It has been shown to be associated with both high BMI and nonalcoholic fatty liver disease in previous study^19^.

Sphingomyelin is found mainly in membranous myelin sheaths and is suggested to be an insulator of nerve fibers. It has been discovered that it plays a role in cell signaling pathways, including apoptosis^20^. Excessive sphingomyelin was previously shown to be associated with insulin resistance^20^.

Sphingosine and sphingadiene are the backbone of all sphingolipids. They and their derivatives are important second messengers involved in functions such as cell growth, differentiation, and apoptosis.

The negative association between sphingolipids and systolic orthostatic blood pressure decrease may possibly be explained by their previously shown association with high BMI, nonalcoholic fatty liver disease and insulin resistance^20,21^. In addition, recent studies have shown that the sphingolipid plasma S1P (Sphingosine-1-Phosphate) links to hypertension and biomarkers of inflammation and cardiovascular disease^22^. S1P has been shown to be released by circulating cells such as leukocytes, erythrocytes and blood platelets facilitated among others by the influence of inflammatory mediators such as TNF-alpha. In our study S1P wasn´t shown to associate with orthostatic blood pressure responses when adjusting for age, gender and supine systolic blood pressure. The role of sphingolipids in cell processes and sphingomyelin’s role as a nerve insulator could on the other hand be regarded as protective features against orthostatic hypotension.

Dihomo-linoleoylcarnitine was another metabolite associated with systolic orthostatic blood pressure response. Previous findings on dihomo-linoleic acid, obesity and metabolic health in humans are mixed^23^. In rodent studies, beneficial effects of dietary linoleic acid administration were found on insulin resistance and glucose tolerance^24^. Long-chain acyl fatty acid derivatives accumulate in the cytosol and serum of patients suffering from mitochondrial carnitine palmitoyltransferase II deficiency, the most common inherited disorder of lipid metabolism in adults. Carnitine plays a critical role in energy production. It transports long-chain fatty acids into the mitochondria so they can be oxidized to produce energy. It also transports the toxic compounds generated out of this cellular organelle to prevent their accumulation. Given these key functions, carnitine is concentrated in tissues like skeletal and cardiac muscle that utilize fatty acids as a dietary fuel^25^.

We found one previous study about metabolic changes preceding cardiac dysfunction that showed an association between linoleoylcarnitine and hypertension and hypertension-induced left ventricular hypertrophy in rodents^26^.

In our study dihomo-linoleoyl carnitine we found a positive association with systolic orthostatic blood pressure decrease.

1-myristoyl-2-arachidonoyl-GPC is a metabolite of the phosphatidylcholine (PC) metabolism. Not much is known about this molecule, whereas three other metabolites of this pathway choline, trimethylamine N-oxide (TMAO), and betaine respectively, previously showed to predict risk for CVD^11–15,25^. Phospholipid synthesis has been shown to be vital for cell survival, normal development and the maintenance of health. Alterations in phospholipid levels have previously been shown to be associated with lipid profiles, obesity and insulin resistance^20,27,28^and with hypertension as well as hypertension-induced left ventricular hypertrophy in rodents^26^.

We found 1-myristol-2-arachidonoyl-GPC to be inversely associated with systolic orthostatic blood pressure decrease.

### Amino acid metabolites in relation to orthostatic blood pressure reactions

In our study, three amino acids and one derivative showed association with systolic orthostatic blood pressure responses.

Creatine is an amino acid that is most abundant in muscles, the heart and brain. Its primary role is to bind with inorganic phosphate in the cell to form phosphocreatine, and thereby serve as a high-energy phosphate source of energy to resynthesize adenosine triphosphate (ATP) that has been degraded to adenosine diphosphate (ADP) + Pi as a source of energy to fuel cellular metabolism. It increases cellular energy availability and is among other consumed by bodybuilders for muscle gain. It has also shown to be neuroprotective and is thought to have anti-inflammatory and immunomodulating effects^29^.

In our study creatine had a negative association with systolic orthostatic blood pressure responses.

Cysteine-S-sulfate is an abnormal metabolite discovered in the urine and blood of a patient with cysteine oxidase deficiency, a rare disorder of sulfur amino acid metabolism associated with brain damage and mental retardation^30^. We found it to be inversely associated with systolic orthostatic blood pressure decrease.

Glutamine is the most abundant and versatile amino acid in the body. In health and disease, the rate of glutamine consumption by immune cells is similar or greater than glucose. For instance, in vitro and in vivo studies have determined that glutamine is an essential nutrient for lymphocyte proliferation and cytokine production, macrophage phagocytic plus secretory activities, and neutrophil bacterial killing. Glutamine release to the circulation and availability is mainly controlled by key metabolic organs, such as the gut, liver, and skeletal muscles^31^. During catabolic/hypercatabolic situations glutamine can become essential for metabolic function. Symptoms of glutamine deficiency include increased susceptibility to infections, bowel changes, diarrhea, ulcers, and weight loss. A side effect of too high levels are vegetative symptoms, skin rash, muscle and joint pain and swelling of hands and feet.

We found glutamine and the glutamine degradant to have a positive association with systolic orthostatic blood pressure decrease.

### Other metabolites in relation to orthostatic blood pressure reactions

Metabolites of the glucuronidation of piperine pathway are a black pepper constituent which recently have shown to have antioxidant, anti-inflammatory, antidiabetic, anti-mutagenic, tumor-inhibiting, drug metabolism inhibiting and antidiarrheal properties^32,33^ in humans. Blood pressure lowering effects were previously reported in animal experiments^34^.

We found glucuronide of piperine metabolite C_17_H_21_NO_3_ to have a negative association with systolic orthostatic blood pressure responses.

### Limitations

This is an exploratory study, meaning that we needed to apply correction for multiple testing. We chose the Bonferroni approach, which may be too conservative, however lowering the risk of type 1 error^18^. The exploratory nature of our study also means, that the results should be confirmed in independent samples, and that the epidemiological and clinical impact of the results is yet to be determined. Still, we find the results valuable as a base for further studies.

Our findings are from the general population in the age group 50–64 years and may not be generalized to other age groups. Regarding the metabolome and cardio-metabolic health it would be interesting in future studies to compare the metabolome in young and healthy age groups with middle aged and old age groups. Cardio-metabolic disease develops and progresses throughout a lifetime and may become manifest in the age-group of the current study, at least as subclinical disease^7,8^. An increased prevalence of orthostatic hypotension is seen after the 6th decade and it is rising exponentially in the last decades of life^1^.

We analyzed possible associations between orthostatic blood pressure responses as a marker of suggested autonomic dysfunction and 818 metabolites to cover as many pathways as possible. We only examined every metabolite for itself, so we did not test for any patterns of differences in clusters of metabolites.

It may be discussed, whether or not the subtle changes in orthostatic blood pressure adaptations, seen in this generally healthy population, reflects overt autonomic dysfunction. However, postural change in blood pressure is one of the five Ewing's tests, which have been used for assessing autonomic function for several decades^35^. Impaired orthostatic blood pressure reactions^36^ as well as deep breathing tests^37^ have previously been shown to be associated with coronary artery calcium deposits in SCAPIS.

The cross-sectional design limits the interpretation of the results: We cannot comment on the causes and effects of single metabolites on orthostatic blood pressure responses, but solely that an association exists. It is however known, that autonomic dysfunction in most cases is a consequence of cardio-metabolic disease.

A methodological limitation is, that the orthostatic blood pressure responses were only measured once at 3 min after standing up, and there are no additional measurements to record possible delayed abnormal orthostatic blood pressure responses. The low prevalence of manifest orthostatic hypotension is in concordance with the prevalence found in earlier studies^1^. Nevertheless, the low prevalence is a limitation and it is hard to predict the reliability of just a single visit evaluation. Orthostatic hypotension is known to possibly be affected by acute and transient conditions like dehydration, hypoglycemia, fatigue and low blood count^1,3^. The reliability of single orthostatic blood pressure recordings should be assessed in future studies. It should also be mentioned, that more precise measurement of orthostatic blood pressure reactions, including the detection of transient orthostatic hypotension, can be achieved by a tilt-test with prolonged continuous blood pressure and heart rate monitoring in up to 20 min after tilting^2^.

Another limitation is, that we in this exploratory study didn´t adjust for kidney and liver function and other conditions that could possibly influence the absorption, excretion, accumulation and half-life of the metabolites, and thereby the levels of measured concentrations.

On the other hand, we managed to include a vast amount of metabolites in the analyses. The real effects of the metabolites found to associate with orthostatic blood pressure responses should now be addressed more thoroughly in future studies.

Finally, since we assessed linear models in this study, we cannot rule out that some metabolites could be non-linearly related to orthostatic blood pressure reactions.

## Conclusions

In this exploratory study we found 22 metabolites, of which nine lipids, that were associated with orthostatic blood pressure responses. Of interest, eleven of these metabolites, including six lipids, four amino acids and one plant derivative, associated with orthostatic blood pressure responses after additional adjustment for traditional metabolic cardiovascular risk factors.

Whereas the clinical importance of our findings is currently limited, we suggest, that these eleven metabolites and their pathways should be further investigated in order to enhance the understanding of the role of the metabolome in the association between cardiovascular disease and autonomic dysfunction.

## Perspectives

Impaired orthostatic blood pressure response is a very common phenomenon that increases with age. Even though this condition may often be asymptomatic, even a slightly impaired blood pressure response to standing may reflect underlying cardiovascular autonomic dysfunction, and such abnormalities are strongly associated with cardiovascular risk factors and incident cardiovascular disease.

The metabolome is believed to harbor an enormous pool of novel valuable information on cardiovascular disease. Few studies have however investigated the human metabolome in relation to autonomic dysfunction. In this study, we identified eleven metabolites, that associated with impaired systolic orthostatic blood pressure reactions in a middle-aged population-based cohort.

These eleven metabolites seem to be involved in diverse pathways, including metabolism, neuroprotection, inflammation and/or immunomodulation. Whereas based on an early exploratory study, we think that our current results may still guide future research of the human metabolome in relation to cardiovascular autonomic dysfunction. Ultimately, such studies may reveal new pathways that could be targeted for treatment.

## Novelty and relevance

What is new:

1. This study reveals eleven diverse metabolites that are independently associated with impaired orthostatic blood pressure reaction.

What is relevant:

1. An impaired orthostatic blood pressure reaction is a marker of subtle cardiovascular autonomic dysfunction, that are associated with cardiovascular risk. We here point to eleven specific metabolites that may be involved in this association.

Clinical and pathological implications:

1. Our results may guide future research on the human metabolome in relation to cardiovascular autonomic dysfunction. Ultimately, such studies may reveal new pathways that could be targeted for prevention and treatment of cardiovascular disease.

### Supplementary Information

Supplementary Information 1.

Supplementary Information 2.

Supplementary Information 3.

## Data Availability

The datasets generated during and/or analyzed during the current study are not publicly available due to regulations/IRB but are available from the corresponding author on reasonable request.

## Abbreviations

OH: Orthostatic hypotension
CVD: Cardiovascular disease
LPS: Lipopolysaccharides
TMAO: Trimethylamine oxide
SCFA: Short chain fatty acids
BCAA: Branched-chain amino acids
AAA: Aromatic amino acids
SCAPIS: The Swedish CardioPulmonary bioImage Study
RP: Reverse phases
UPLC: Ultrahigh performance liquid chromatography-tandem
MS: Mass spectroscopy
ESI: Negative ion mode electrospray ionization
SD: Standard deviation
CI: Confidence interval
DHSM: Behenoyl dihydro-sphingomyelin
S1P: Sphingosine-1-phosphate
PC: Phosphatidylcholine
ATP: Adenosine triphosphate
ADP: Adenosine diphosphate
