# Supplementary material for: The relationships between the plasma metabolome and orthostatic blood pressure responses
Source: Sci Rep. 2023 Oct 25;13:18244. doi: 10.1038/s41598-023-44226-z (PMC10600108; doi:10.1038/s41598-023-44226-z)
Supplement: Supplementary file 1 — Supplementary Information 1. [file 41598_2023_44226_MOESM1_ESM.docx]

**Supplementary Table 1: Clinical characteristics in included and excluded subjects**

|  | **Included in all analyses (n=3,803)** | **Excluded (n=2,448)** |
| --- | --- | --- |
| **Orthostatic hypotension  (n, %)** | 56, 1.5 | 50, 2.3; n =2,197 |
| **Age (years)** | 57.42 ±4.27 | 57.60 ±4.31; n = 2,448 |
| **Women (n, %)** | 1,984, 52.2 | 1338. 54.7; n = 2,448 |
| **BMI (Kg/m^2^)*** | 27.17 ±4.41 | 27.52 ±5.03; n = 2,447 |
| **Orthostatic systolic blood pressure decrease (mmHg)** | -4.40 ±10.20 | -2.78 ±10.39; n = 2,197 |
| **Supine systolic blood pressure (mmHg)** | 121.71 ±16.14 | 124.62 ±17.00; n = 2,223 |
| **Resting heart rate (bpm)** | 60.84 ±9.05 | 61.19 ± 9.22; n = 2,403 |
| **Diabetes (n, %)*** | 314, 8,3 | 238, 9.9; n = 2,414 |
| **Smokers (n, %)*** | 548, 14.4 | 417; 17.0 n = 2,448 |
| **Anti-hypertensive drug treatment (n, %)*** | 772, 20.3 | 513; 21.0; n = 2,440 |
| The values were presented as mean ± standard deviation (SD) for continuous variables and numbers, percentages (n, %) for categorical variables. | | |
